# MAGICIAN: MAG simulation for investigating criteria for bioinformatic analysis

**DOI:** 10.1186/s12864-023-09912-2

**Published:** 2024-01-12

**Authors:** Kat Steinke, Sünje J. Pamp, Patrick Munk

**Affiliations:** 1 Center for Genomic Epidemiology, National Food Institute, Technical University of Denmark, Kemitorvet 204, 2800 Kongens Lyngby, Denmark; 2 Department of Clinical Microbiology, Odense University Hospital, J. B. Winsløws Vej 21, 5000 Odense, Denmark

**Keywords:** Metagenomics, Simulation, Benchmarking

## Abstract

**Background:**

The possibility of recovering metagenome-assembled genomes (MAGs) from sequence reads allows for further insights into microbial communities and their members, possibly even analyzing such sequences with tools designed for single-isolate genomes. As result quality depends on sequence quality, performance of tools for single-isolate genomes on MAGs should be tested beforehand. Bioinformatics can be leveraged to quickly create varied synthetic test sets with known composition for this purpose.

**Results:**

We present MAGICIAN, a flexible, user-friendly pipeline for the simulation of MAGs. MAGICIAN combines a synthetic metagenome simulator with a metagenomic assembly and binning pipeline to simulate MAGs based on user-supplied input genomes, allowing users to test performance of tools on MAGs while having a ground truth to compare results to. Using MAGICIAN, we found that even very slight (1%) changes in depth of coverage can drastically affect whether a genome can be recovered. We also demonstrate the use of simulated MAGs by evaluating the suitability of such genomes obtained with MAGICIAN’s current default pipeline for analysis with the antimicrobial resistance gene identification tool ResFinder.

**Conclusions:**

Using MAGICIAN, it is possible to simulate MAGs which, while generally high in quality, reflect issues encountered with real-world data, thus providing realistic best-case data. Evaluating the results of ResFinder analysis of these genomes revealed a risk for plausible-looking false positives, which underlines the need for pipeline validation so that researchers are aware of the potential issues when interpreting real-world data. Furthermore, the effects of fluctuations in depth of coverage on genome recovery in our simulated “random sequencing” warrant further investigation and indicate random subsampling of reads may affect discovery of more genomes.

**Supplementary Information:**

The online version contains supplementary material available at 10.1186/s12864-023-09912-2.

## Background

### Metagenomics and MAGs

In recent years, metagenomic technologies have grown in importance in a wide range of fields, from ecology to healthcare [1]. “Shotgun metagenomics”, the non-targeted sequencing of microbial genomes in a sample, allows the investigation of complex microbial communities by a number of analyses [2].

Analyzing metagenomes through de novo assembly to contigs and binning of those contigs to yield so-called metagenome-assembled genomes (MAGs) has become increasingly common over the course of the last years, with studies assembling large numbers of genomes from metagenomic reads (see for example [3–5]). This approach has already provided a greater overview of microbial diversity and deeper insights into new metabolic pathways [6]. Some have even attempted to analyze MAGs with tools originally designed for single-isolate genomes, for instance by using the genome mining tool antiSMASH to predict potential natural products in a number of MAGs [7]. However, the quality of the results of such analyses depends on the quality of the input (see e.g. [8] for how this applies to antiSMASH). It is thus necessary to establish what level of MAG quality is necessary to yield reliable results from tools designed for isolated genomes. Manually completed and curated MAGs would be ideal when using MAGs as input for single-isolate tools, as even high-quality automatically assembled MAGs have been found to contain errors [6]. However, this is a time-consuming process; with some studies producing as many as 150,000 MAGs, automation is required to feasibly explore the wealth of available data. It therefore is necessary to evaluate errors of MAG generating pipelines and their effects on results from single-isolate tools.

### Simulating MAGs: motivation and methods

“Real-world” data is commonly used in benchmarking and optimization studies for genome assemblers and binners, as seen for example in a study by Papudeshi et al. [9] comparing three assemblers and two binners. This has the advantage of reflecting the complexity of the data processed with such programs, but means the underlying composition of the community used for testing remains unknown [10, 11]. When evaluating the performance of tools meant for single-isolate WGS rather than metagenomes on MAGs, the issue becomes even greater - in some cases, assembly-free methods provide some basis for comparison, but it is difficult to confirm the results of analyses where genomic context is relevant.

For such purposes, a potential alternative may be the use of synthetic metagenomes - a term covering both approaches in which a community is simulated by pooling DNA from various organisms in vitro and sequencing it (e.g. the MBARC-26 dataset [12]), and ones in which existing, complete genomes are used to simulate metagenomic reads in silico (e.g. the data used in the CAMI challenge [13]). With these synthetic metagenomes, the composition of the community and the genomes of the organisms comprising it are already known; this is especially helpful in examining the performance of single-isolate tools on MAGs, as one can obtain a point of comparison by analyzing the original sequences with the tool in question. The approach is also highly flexible - one can fine-tune community composition to suit any specific question, and for in silico synthetic metagenomes, changing a few lines of code may be all that is required to change sequencing technology. A number of standalone read simulators emulating different sequencers can be employed to simulate metagenomic reads, or one may use pipelines such as CAMISIM [14], a tool originally developed for the aforementioned CAMI challenge that can utilize multiple read simulators in order to simulate Illumina, Nanopore, PacBio and error-free reads, or Tamock [11], which recreates the taxonomic composition of existing metagenomic samples with reads generated from reference genomes. However, no similarly flexible tool for the simulation of MAGs has been published; we therefore present MAGICIAN, a user-friendly pipeline for MAG simulation for a wide range of purposes.

### Antimicrobial resistance monitoring through metagenomics - a case for MAGs?

One challenge which may be addressed by metagenomics is the rise of antimicrobial resistance (AMR) - a global challenge which threatens to make the treatment of even common infections a matter of chance [15]. To overcome this threat, metagenomic methods have been employed to monitor the spread of AMR, up to and including the global surveillance of antimicrobial resistance through metagenomic analysis of sewage, as proposed in a recent study examining samples from 60 countries [16], or other environmental samples such as samples from public transit systems [17]. Using a read-based approach, the study of Hendriksen et al. [16] was able to make important observations on the global prevalence and distribution of antimicrobial resistance genes (ARGs). Yet more information could be gained by complementing this with an assembly-based approach - in addition to identifying what genes were found in a sample, MAGs would allow to place them into context, thus revealing e.g. whether a certain ARG was present due to the outbreak of a resistant pathogen or coincidentally occurred in a commensal organism, or whether a large number of ARGs belonged to many organisms or a few multiresistant “superbugs”. As valuable as the information from this may be, however, errors in such analyses could have severe consequences - a wrongly assembled MAG could mean that a potential outbreak of a multiresistant pathogen is dismissed as a coincidental find in a commensal. We therefore decided to test the performance of a commonly used tool for AMR detection aimed at a broad range of people, ResFinder 4.0 [18], on MAGs to uncover any potential issues.

## Materials and methods

**Fig. 1:**
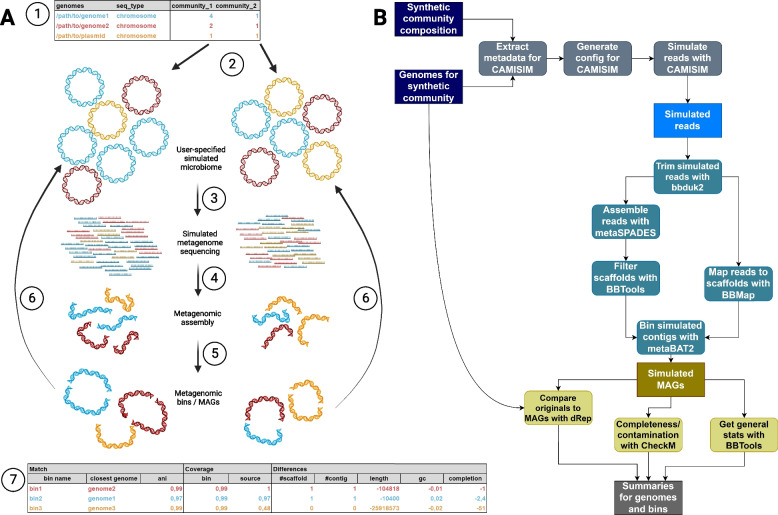
**A** Diagram of the process in the simulated experiments; 1: the user supplies genomes and their required relative abundances for one or more community samples. 2: Microbiomes with the user-supplied compositions are simulated. 3: Shotgun metagenomic sequencing of the microbiomes is simulated resulting in reads with the chosen error profile. 4: Metagenomic assembly is performed on each microbiome. 5: Genomic binning is performed in an attemt to group the contigs/scaffolds into original input genomes. 6: The metagenomic bins are compared to the user-supplied input genomes. 7: Summary tables are provided to the user showing matched pairs of genomes and bins, how well they cover each other and differ in terms of fragmentation, % GC, length, and completion. Note that only a single summary table corresponding to community_1 (left) is shown. **B** A detailed overview of the workflow of MAGICIAN, from generation of configuration files for CAMISIM to summarizing quality control measures, showing the software used. Created with BioRender.com

As shown in Fig. 1A, MAGICIAN generates synthetic MAGs by first simulating reads from a metagenomic sample composed of specified organisms, assembling these reads, and binning the resulting contigs. Afterwards, various measures such as N50, L50 and similarity to input genomes are generated. The workflow is implemented using Snakemake [19], as this allows for a great degree of flexibility as well as a user-friendly way of handling dependencies by running each step of the workflow in a dedicated conda environment.

A detailed overview of the workflow including the tools used can be seen in Fig. 1B.

### Input

To generate MAGs using MAGICIAN, sequences comprising an in silico “metagenomic community” (in FASTA or GenBank format) are required. The user supplies these through a comma-separated overview table containing the path to each sequence, information on whether it is a chromosome or plasmid sequence, as well as the relative amount of genome copies of the sequences in each “community” simulated (“relative abundance” for brevity, following the terminology of CAMISIM [14], the metagenome simulation pipeline used). An example of such a table is seen in Table 1.

**Table 1:** Sample input table for the MAGICIAN pipeline

genomes	seq_type	sample_1	sample_2
/path/to/seq_1.fa	chromosome	1	2
/path/to/seq_2.gbk	chromosome	1	1
/path/to/seq_3.gbk	plasmid	1	3

MAGICIAN is then run through a Python script which takes the overview table as input. The pipeline defaults to running the entire workflow, but desired target file or rule can be specified, as with a Snakemake workflow; error profiles used for read simulation (see below) can be specified, and commands may be passed through to Snakemake itself. A configuration file is used to specify what environment manager to use as well as the location of CAMISIM (see below).

### Read simulation

The metagenome simulation pipeline CAMISIM [14] is used to simulate the initial metagenomic sample. Specifically, Illumina reads are simulated by using the read simulator ART [20] within CAMISIM; to allow users to simulate reads from other sequencers than those already included with CAMISIM, a version of CAMISIM which is able to use user-supplied error profiles with ART is required. In the current version of MAGICIAN, this is implemented through a fork of CAMISIM (code available at https://github.com/KatSteinke/CAMISIM), a functionality that has since been integrated in CAMISIM with version 1.3. CAMISIM simulates forward and reverse reads for each genome individually; to obtain a “metagenomic dataset”, reads are then pooled to obtain two files respectively containing all forward and all reverse reads.

### MAG creation

To obtain synthetic MAGs, the pooled reads are processed with a workflow that closely follows realistic workflows:

- reads are trimmed with BBDuk2 [21]
- trimmed reads are assembled using metaSPAdes [22]
- assembled scaffolds are filtered by size using BBMap’s rename.sh script [21]
- reads are mapped back to the filtered scaffolds using BBMap; SAMtools [23] is then used to convert the mapped reads to BAM format and sort them by coordinates, and finally a depth file giving depth of coverage for each scaffold is generated with the jgi_summarize_bam_contig_depths tool included with MetaBAT2 [24]
- scaffolds are binned using MetaBAT2; in simulated communities that contain plasmids, minimum bin size is reduced to avoid excluding all but the largest plasmids

### Output

The primary output of MAGICIAN consists of the synthetic MAGs simulated on the basis of the supplied genomes. To evaluate how closely these match the source genomes, MAGICIAN also performs a number of analyses:

- contig and scaffold statistics are obtained with the statswrapper.sh script in BBMap [21], so that issues such as substantial fragmentation or large differences in GC content can be identified.
- taxonomy, completeness and contamination are estimated using CheckM [25]
- as the original sequences are known, the synthetic MAGs are compared to these using dRep compare [26], which allows for a direct evaluation of sequence similarity between synthetic MAGs and original sequences using average nucleotide identity (ANI). The pipeline uses dRep compare’s default ANImf algorithm, which calculates ANI and clusters based on Nucmer [27] alignments which are filtered to only include the longest consistent alignments for reference and query respectively.

Results of these analyses are then summarized to give the user an overview of the relevant statistics. This includes both the statistics described above for bins and original genomes in a “general summary” table, and a comparison of each bin to the original sequence to which it has the highest ANI in a “bin summary” table. Examples of such tables are given as Additional files 1 (general summary) and 2 (bin summary), taken directly from the pipeline’s output for one replicate of a MAGICIAN run with a simulated microbial community used in this work (community 1a, described below under “Constructing synthetic metagenomes for testing”).

### Code availability

The code of MAGICIAN can be found at https://github.com/KatSteinke/magician (https://doi.org/10.5281/zenodo.10144427).

### Constructing synthetic metagenomes for testing

In order to test the performance of MAGICIAN, a collection of metagenomic samples consisting of 17 organisms (see Supplementary Table 1) was simulated as detailed below. Organisms comprising the community were selected to prioritize phylogenetic diversity (with organisms sharing different taxonomic levels), as well as diversity in GC content and genome size. Both common laboratory strains and clinically relevant organisms such as the ESKAPE pathogens [28] were included. To evaluate whether assembly and binning issues caused by the presence of closely related organisms could be reproduced by MAGICIAN output, several different samples containing one or more close relatives of organisms in the community were added. These formed community 1a (with multiple related organisms) and community 1b (with a single pair of related organisms). Some of the organisms selected also carried plasmids - specifically, *Acinetobacter baumannii* XH386, *Bifidobacterium choerinum* FMB-1, *Enterobacter cloacae* EN3600, *Enterococcus faecium* isolate Ef_aus00233, and *Salmonella* Typhimurium 81741. Plasmid sequences were not included in the original community, but a separate community (“community 2”) which included these sequences were simulated in order to examine the effects of the presence of plasmids.

Quite surprisingly, seemingly small variations in read simulation even of abundant organisms substantially affected binning, to the point of genomes no longer being reconstructed. Simulations were repeated in triplicate to compensate for this, with the exception of the “baseline” community, which was simulated five times in order to investigate the issue.

To obtain the baseline community, the relative amount of copies for each organism in the community (“relative abundance” in CAMISIM) was initially generated by randomly drawing from a uniform distribution between 1 and 3.5, then manually adjusting the distribution to avoid organisms not being recovered due to low abundance as well as “hybrid bins” arising from related organisms with too similar abundance, so that the baseline represented a synthetic “best case” to which issues could then be introduced systematically. However, due to the abovementioned effect of random variation, recovery of all source organisms could not be consistently achieved.

### Investigating failure to recover source organisms

In order to investigate the effect of variations in depth on recovery of source organisms further, reads from one replicate of simulated community 1a in which contigs from one organism (*B. cereus*) could not be binned were randomly downsampled in triplicate, retaining 99% of the reads. This was performed with sorted .bam files, the final step before calculating depth, using reformat.sh from the BBTools suite [21]:
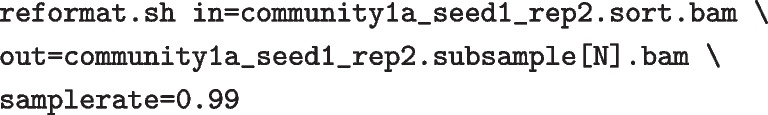
 Depth was then recalculated using the downsampled files in the same manner as in the MAGICIAN pipeline proper. Binning was then performed with MetaBAT2 once more, using the same settings (including the random seed for MetaBAT2) as in MAGICIAN, but with the depth files obtained with the downsampled reads. Finally, bins obtained in this manner were compared to the original genomes using dRep compare with the settings used in MAGICIAN to investigate whether scaffolds from the “missing” organism could be binned.

### Matching bins to original genomes

To compare the simulated MAGs to the sequences they were derived from, each source sequence (genome or plasmid) was assigned at most one bin for ease of analysis. As a bin also could match multiple source sequences, it was also necessary to ensure that each bin was only assigned to at most one source sequence. A bin was therefore only assigned to a source sequence if a) the bin was the bin with the highest ANI to the source sequence, as determined by dRep compare, b) the source sequence was the sequence with the highest ANI to the bin, as determined by dRep compare, and c) this bin was not simply the one that contained all unbinned scaffolds. Throughout this work, when discussing matches between bins and source sequences, this was the method applied, unless stated otherwise. For brevity, bins that have been matched to a source sequence in this way will generally be referred to by the source sequence’s name in figures unless explicitly noted otherwise. To aid in this process, MAGICIAN reports the closest source sequence for each bin (criterion b)) in one of the output files produced, the “bin summary” table. An example of such a table is included as Additional file 2.

### Identifying contamination

As the original genomes were known, contamination could be accurately calculated rather than estimated, through the alignment of input sequences to output bins. To examine alignments in detail, alignment was performed using Nucmer version 3.23 [27] at default settings: 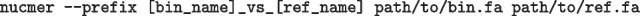 and vice versa. Subsequently, hits were filtered using show-coords to only include matches with a length of at least 2000 basepairs and 70% identity: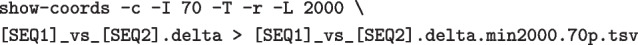 For comparison, the table of results was then filtered further in Python using the pandas library to obtain only hits with 100% identity.

### Performance of a single-genome tool: ResFinder

ResFinder 4.0 [18] was run on FASTA files of the original genomes as well as the simulated MAGs. As one test dataset included two *Salmonella* Typhimurium strains differing in the presence of a point mutation conferring resistance, “Salmonella” was selected as the taxa for the purpose of identifying point mutations. It was first established which ARGs (and mutations in the case of *Salmonella*) were found by ResFinder in the original sequences used as a basis for the synthetic metagenome. AMR predictions were obtained for all 84 antimicrobials and antimicrobial combinations available in ResFinder. Subsequently, the MAGs reconstructed from the different “samples” were analyzed with ResFinder and results were compared. For evaluating the results, true and false positives and negatives were defined by whether predicted antimicrobial resistances and susceptibilities in a MAG matched these of the original genome it matched, where this was applicable; the organism’s true AMR profile was not considered. From these observations, sensitivity, specificity, accuracy, precision and F-score were calculated for each simulated metagenomic community.

### Statistical analysis

Statistical analysis was performed in R.

## Results

### Genome retrieval and factors affecting it

**Fig. 2:**
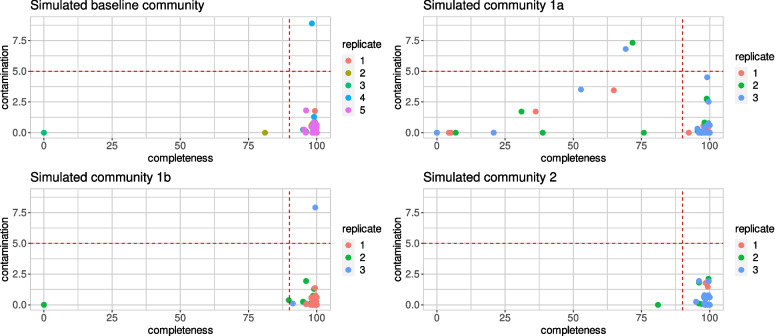
Completeness and contamination values calculated by CheckM for the baseline community, communities with related organisms, and communities with plasmids, colored by replicate. Dotted lines represent cutoffs for contamination and completeness for high-quality genomes. Plotted in R using ggplot2

As an initial demonstration of MAGICIAN’s core functionality, a microbial community consisting of 17 organisms was simulated. Metagenome composition was subsequently varied to explore their impact on the MAGs the pipeline produced. As shown in Fig. 2, in the baseline community, the majority of bins fulfilled Bowers et al.’s completeness and contamination cutoffs for high-quality genomes (over 90% completeness, under 5% contamination) when using CheckM [29]. In simulated community 1a, containing multiple related organisms, multiple bins failed the completeness and/or contamination thresholds, while in simulated community 1b, with only one pair of related organisms, issues were only visible in two out of three replicates. The addition of plasmids in simulated community 2 did not appear to affect completeness and contamination scores, likely because plasmids were not recovered in the first place.

When identifying genome completeness and contamination by aligning bins to the original genomes using Mummer, a similar pattern is visible (see Supplementary Table 2). However, in some cases contamination appears far more substantial than detected with CheckM. As expected, related organisms often are the source of the contamination, such as *K. pneumoniae* sequences being found in a bin primarily matching *Enterobacter cloacae*.

An additional finding which can be seen in Fig. 2 is that relatively small differences in simulated reads - specifically, a different random seed - substantially affected the resulting bins. Despite all other parameters being identical, simulations of the baseline community led to a substantially more contaminated bin in only one replicate, and incomplete bins in two. The issue becomes even more apparent upon matching bins to source genomes - in several simulations of the baseline community, no bin matching *B. cereus* ATCC 14579 was found, while dRep compare showed the closest match to *B. cereus* ATCC 14579 was the unbinned contigs. This may for instance be seen in the dRep results of Additional file 1, and the source-to-bin matches in Additional file 2. No obvious differences in read quality or coverage of the genome were apparent between replicates, however (see Supplementary Fig. 1), so that no cause for the issue was apparent. To investigate the effects of small fluctuations in depth, one dataset in which *B. cereus* scaffolds failed to be binned was subsampled to retain 99% of the reads in three replicates; in these, *B. cereus* scaffolds could be binned (see Additional file 3 for dRep compare’s Nucmer-based Ndb.csv output for all three replicates).

### Performance of ResFinder on simulated MAGs

To demonstrate the utility of simulated MAGs in evaluating whether a given assembly and binning pipeline results in metagenomic assemblies of sufficient quality for use with a given tool, MAGs reconstructed from the various simulated samples were then analyzed with ResFinder, a tool used to predict AMR. In general, F-scores seemed to indicate acceptable sensitivity and specificity, ranging from 0.74 to 0.84. As expected, ResFinder performed worse for the simulated communities introducing known issues with MAG assembly; when multiple closely related organisms were added (community 1a), F-scores worsened significantly compared to the baseline, though a trend may be visible for the other communities (see Supplementary Table 3).

#### Challenges encountered in individual genomes

**Fig. 3:**
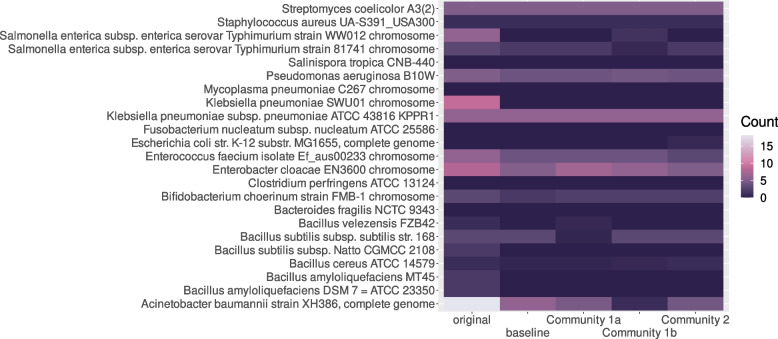
Heatmap showing the absolute amount of AMR genes in each organism (x-axis) or its matching bin by community (y-axis), averaged across replicates. Gene counts in the original sequences are shown in the leftmost column. Only chromosomal sequences were included as plasmids were too rarely recovered to add meaningful information. The plot was created in R using ggplot2

While examining results on a community level did not suggest drastic differences between results obtained with the original sequences and with the reconstructed MAGs, this could vary substantially on an individual level. This is visible in Fig. 3, showing ARG counts averaged across triplicates for each organism in each simulated community. For instance, while the original genome of *Acinetobacter baumannii* XH386 was predicted to contain 18 ARGs, the MAGs in the baseline community contained only six of them. Most commonly, as in the case of *A. baumannii* XH368, the issue was that not all ARGs present in a sequence could be found in the corresponding bins. With the introduction of plasmids, shown in simulated community 2, further issues appeared. As seen in Fig. 3, *Escherichia coli* K-12 was not predicted to contain any ARGs; however, when low copy-number plasmids were added to the simulated community, MAGs matching *E. coli* K-12 were predicted to contain the *bla*CTX-M-3 gene, which encodes the extended-spectrum beta-lactamase blaCTX [30], in two out of three replicates. From the original non-metagenomic sequences, we can see this gene actually originates from the *Enterobacter cloacae* EN3600 plasmid unnamed6.

## Discussion

### Applicability of MAGICIAN for MAG simulation

MAGICIAN provides a convenient way to simulate entire modern, genome-focused metagenomic studies in silico. Most MAGs simulated in this study fulfilled completeness and contamination requirements for high quality draft genomes as defined by Bowers et al. [29]; while this of course is not the case for all MAGs obtained in real studies, many such studies result in so many MAGs that further investigation could easily be limited to high-quality genomes while still obtaining large amounts of data. Based on the “baseline” community, metagenomic communities were put together as “test cases” provoking a range of issues occurring in real metagenomic samples. The introduction of closely related organisms in communities 1a and 1b led to decreased completeness and increased contamination due to relatively highly fragmented bins and “hybrid bins” caused by metaSPAdes’s attempts to prevent this fragmentation through “consensus assemblies” [22]. Both of these issues have been found to occur in studies of real MAGs [6]. Plasmids were generally not recovered; this, too, is consistent with the challenges posed by plasmids in real-world metagenomic samples [31, 32]. However, the presence of plasmids may still affect the assembled MAGs and thereby downstream analysis, as plasmids may be binned with entirely wrong organisms. For optimizing MAG quality, one might therefore consider actively removing the circular plasmid molecules before sequencing.

However, MAG quality and reconstruction was also affected by another factor: the random seed used in read simulation with ART. With all other parameters in the pipeline unchanged, different random seeds for read simulation resulted in substantial differences in the recovery of MAGs, with scaffolds from *B. cereus* failing to be binned on multiple occasions. No differences in read quality were evident using FastQC, and when examining mappings of the generated reads to the source genome, no obvious anomalies occurring only in one of the two groups were evident. The presence of scaffolds belonging to *B. cereus* in the assemblies produced by metaSPAdes was also verified. No anomalies were apparent. Subsampling one read set which produced such binning issues to retain 99% of reads led to successful binning of the scaffolds from the “missing” organism, suggesting that small fluctuations in depth seem to have large and surprising effects on the ability to recover genomes. While further investigation would have exceeded the scope of this work, it is clearly warranted in the future, to verify whether genome binning success is also this sensitive in real datasets. That could suggest even a slight sub-sampling of real read sets could help discover many more previously missed MAGs and we hope MAGICIAN will help shape and pose such future hypotheses.

### Use of simulated MAGs: testing single-genome tools

To demonstrate how simulated MAGs generated by MAGICIAN could help evaluate the performance and pitfalls when using whole-genome tools on MAGs, we compared ResFinder AMR predictions of MAGICIAN input genomes and output bins.

While results generally appeared promising on a community level, individual organisms posed a number of issues, with large numbers of ARGs overlooked or in some cases even mis-binned to wrong, but plausible genomes.

#### The problem with plasmids: a plausible-looking false positive

In the simulated microbial community containing low-copy number plasmids, MAGs matching *E. coli* K-12, an organism without any ARGs in ResFinder, were found to contain the extended-spectrum beta-lactamase gene *bla*CTX-M-3. The incorrectly binned MAGs would not have aroused immediate suspicion: they were not unusually fragmented, and their genome size, matching that of the original genome, was not inconsistent with CheckM placing them within *Enterobacteriaceae*. Without knowledge of the underlying “true” sequence, a researcher faced with the ResFinder prediction of *bla*CTX-M-3 in an organism placed within Enterobacteriacea by CheckM would now likely use their knowledge of ARGs in order to investigate the result, checking for irregularities such as atypical ARGs or acquired resistance to an antibiotic the organism is intrinsically resistant to. However, in this case, the result would still be plausible, as *bla*CTX-M-3 is indeed found in *Enterobacteriaceae* [30]. Some questions may be raised by the gene being plasmid-borne, but these too have a plausible answer indicating a far smaller error: tetranucleotide frequency, one of the factors used by MetaBAT2 in binning contigs [24], can be similar in plasmids and their hosts [33] - absent the underlying sequences, this might indicate a low copy number plasmid being binned with its host, rather than an entirely different organism. Thus, the researcher could be led to accept ResFinder’s prediction and identify a common laboratory strain of *E. coli* as a potential ESBL-carrying pathogen. This clearly underlines the importance of using simulated MAGs whose underlying sequences are known in initial investigations of the performance of tools designed for single-isolate genomes on MAGs. Though the “ground truth” of course is not known for real metagenomic samples, knowing the types of errors occuring in MAGs should inform which conclusions are permissible.

#### Use of simulated MAGs: perspectives

In the present work, we showed the utility of MAGs created by MAGICIAN in evaluating the suitability of MAGs obtained with a given pipeline for analysis with a given single-isolate tool and investigating the impact of simulated metagenome composition on the results. Beyond this, MAGICIAN could also be used to simulate test datasets for use in developing and/or testing tools specifically designed to work with MAGs; while the MAGs simulated here tend to err on the side of high quality, they do provide a “best case” to test against, and future versions of MAGICIAN may allow the combination of real-world reads with “spiked-in” simulated data.

Using MAGICIAN, metagenomic data from any step of a metagenomic assembly workflow, from reads to entire MAGs, can be simulated for a range of purposes. In the present work, MAGs simulated with MAGICIAN have been used to evaluate how one tool developed for single-isolate genomes performed on MAGs obtained with a specific pipeline and how the composition of the simulated metagenome impacted the results of the tool. The suitability of other pipelines both for this and other tools may be investigated in the future - different tools have different requirements, which may pose different challenges to MAG retrieval. As tools specifically designed to work with MAGs are developed, synthetic MAGs may also provide easily generated test sets to aid in this, as they tend to have the same issues as realistic data.

### MAGICIAN: perspectives

The potential versatility of the Snakemake-based pipeline is far from exhausted. Further additions to the pipeline could make it more flexible by allowing the user to choose between e.g. read simulators, assemblers, binners and performing plasmid-specific assembly. More complex sequencing strategies such as the combination of short and long reads could also be simulated. This technique shows great results in modern hybrid assemblers like OPERA-MS [34]. Being able to select the right combination of instrument and sequencing depth could greatly increase the value of the final datasets. To better reflect the complexity of real metagenomic samples, an option to “spike” an existing sample with simulated reads could also be introduced.

## Conclusions

MAGICIAN makes it possible to simulate metagenomic reads and MAGs from user-defined synthetic communities in silico and easily vary the composition of these communities and simulate large numbers of experiments. This allows users to investigate a wide range of questions and gain an estimate of best-case performance of the assembly/binning pipelines used and the suitability of results for downstream analysis tools before any sequencing is performed. To demonstrate this functionality, MAGs from a range of synthetic communities obtained by systematically varying an initial “baseline” community composition through introduction of related organisms and plasmids, as well as varying sequencing parameters, were simulated. It was subsequently evaluated how well MAGs could be recovered under the specific conditions, and whether this was biologically plausible. Results generally resembled what could be expected from reality: plasmids could generally not be recovered, and reconstructing closely related organisms was challenging. MAGs simulated in this work in fact demonstrated two common issues occurring with closely related organisms: in most cases, bins containing sequences from the related organisms were highly fragmented, but when using a pair of highly similar strains of *Salmonella* Typhimurium, metaSPAdes instead was able to create longer scaffolds based on combining the two, avoiding excessive fragmentation but potentially creating chimeric sequences.

In certain edge cases, two samples simulated from the same underlying community composition, with the same read simulator with the same settings may result in substantially different results after binning, with one of the members of the synthetic community being unable to be reconstructed in one sample while being found without issues in the other, even though there were only slight differences in read simulation. This may warrant further investigation in vitro, as this may reflect an important and arbitrary issue in real-world metagenomic datasets.

The use of simulated MAGs for investigating the suitability of MAGs obtained with a given pipeline for a given tool was also demonstrated through analyzing the AMR profile of simulated MAGs with ResFinder. This showed generally promising results, but also revealed cases of acquired ARGs being wrongly binned to a genome undeserving of the blame. In some cases, these issues could only be identified because the sequences of the underlying community were known. In summary, this work both provides a user-friendly, flexible tool for the simulation of MAGs for a wide range of uses, and demonstrates that it brings considerable advantages to one potential application.

### Supplementary information

**Additional file 1.** General summary of simulated community 1a. MAGICIAN output summarizing general information on one of the simulation runs for community 1a. BBTools’ stats output is summarized in the “BBstats” sheet, CheckM output is summarized in the “CheckM” sheet, and dRep compare output is summarized in the “dRep” sheet. Explanations of terminology are given in the “explanations” sheet.

**Additional file 2.** Bin-focused summary of simulated community 1a. MAGICIAN output comparing bins to the input genomes they have the highest ANI to for one of the simulation runs for community 1a. Comparisons are shown in the “summary” sheet, while explanations are given in the “explanations” sheet.

**Additional file 3.** dRep compare Nucmer outputs for simulated community 1a subsamples. Raw outputs of nucleotide-based ANI comparisons by dRep compare for three replicates of simulated community 1a in which 1% of already sorted and filtered reads have been randomly removed in order to investigate the impact of fluctuations in depth on the ability to bin scaffolds from an organism which could not be binned in the original dataset.

**Additional file 4:** **Supplementary Figure 1.** Coverage of *B. cereus* ATCC 14579 chromosome by reads simulated with CAMISIM in the original test of a fixed seed for MetaBAT 2, the test of fixed seeds for both CAMISIM and MetaBAT 2, and five simulations with a random CAMISIM seed and fixed MetaBAT 2 seed. Read mappings were taken from CAMISIM output and visualized in the alignment viewer Tablet. Black coordinates on the left show the range of the entire overview, red coordinates on the right show the range selected in the red box.

**Additional file 5:** **Supplementary Table 1.** Compositions of the simulated metagenomic communities.

**Additional file 6:** **Supplementary Table 2.** Coverage of original genomes by reconstructed MAGs as determined by Nucmer alignment.

**Additional file 7:** **Supplementary Table 3.** Performance characteristics of ResFinder on simulated MAGs.

## Data Availability

Project name: MAGICIAN Project homepage: https://github.com/KatSteinke/magician Operating system(s): Linux Programming language: Python 3 Other requirements: CAMISIM; conda/mamba; git; Snakemake License: Apache 2.0 Any restrictions to use by non-academics: None Accession numbers of the sequences used in the article can be found in Supplementary Table 1.
