# Supplementary figures and images for: MAGICIAN: MAG simulation for investigating criteria for bioinformatic analysis
Source: BMC Genomics. 2024 Jan 12;25:55. doi: 10.1186/s12864-023-09912-2 (PMC10785454; doi:10.1186/s12864-023-09912-2)

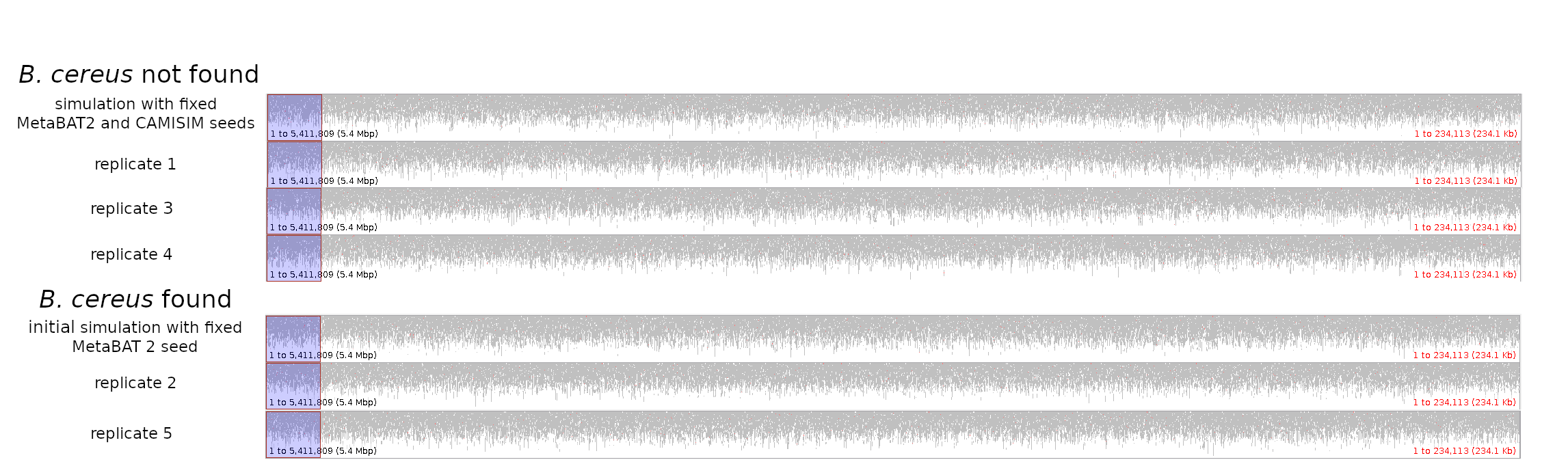

Supplement: Supplementary file 4 — Additional file 4: Supplementary Figure 1. Coverage of B. cereus ATCC 14579 chromosome by reads simulated with CAMISIM in the original test of a fixed seed for MetaBAT 2, the test of fixed seeds for both CAMISIM and MetaBAT 2, and five simulations with a random CAMISIM seed and fixed MetaBAT 2 seed. Read mappings were taken from CAMISIM output and visualized in the alignment viewer Tablet. Black coordinates on the left show the range of the entire overview, red coordinates on the right show the range selected in the red box. [file 12864_2023_9912_MOESM4_ESM.png]
